# Ultrasound-based radiomics combined with B3GALT4 level to predict sentinel lymph node metastasis in primary breast cancer

**DOI:** 10.3389/fonc.2025.1570493

**Published:** 2025-07-11

**Authors:** Yongliang Sha, Song Ge, Yiqiu Wang, Shilong Cai, Chengyi Wang, Huijie Zhuang, Jin Shi, Shiqing He, Xia Sun, Li Ma, Hao Guo, Hui Cheng

**Affiliations:** ^1^ Department of General Surgery, Xuzhou Central Hospital, Xuzhou, Jiangsu, China; ^2^ Department of Ultrasound, Xuzhou Central Hospital, Xuzhou, Jiangsu, China; ^3^ Clinical Medical School, Jining Medical University, Jining, Shandong, China; ^4^ Department of Gynecology and Obstetrics, Xuzhou Central Hospital, Xuzhou, Jiangsu, China

**Keywords:** breast cancer, axillary lymph node metastasis, β-1,3-galactosyltransferase-4, ultrasound radiomics, machine learning

## Abstract

**Objective:**

To evaluate the value of the clinical model for predicting axillary lymph node metastasis (ALNM) of breast cancer before operation by integrating ultrasound (US) and β-1,3-galactosyltransferase-4 (B3GALT4) expression level of the primary tumor.

**Methods:**

A total of 135 breast cancer patients who underwent US examination and axillary lymph nodes dissection (ALND) were enrolled. They were randomly divided into a training group (95 cases) and a verification group (40 cases). The ultrasound imaging characteristics of the primary tumor were extracted from each region of interest (ROI), and the Spearman correlation coefficient, least absolute shrinkage and selection operator (LASSO), and the minimum redundancy maximum relevance (mRMR) were used for feature selection. The radiomics model was constructed by eighteen machine-learning techniques. B3GALT4 expression level of the primary tumor was analyzed using quantitative real-time polymerase chain reaction (qRT-PCR). A clinical model was constructed based on B3GALT4 mRNA level. Further, a nomogram was established by integrating B3GALT4 and the radiomics signature. The effectiveness of each model was evaluated by receiver operating characteristic (ROC) curve, Hosmer-Lemeshow test, calibration curve, and decision curve analyses (DCA).

**Results:**

A total of 1562 radiomics features were extracted, and 30 features were selected. The SVM model had the highest AUC values of 0.937 and 0.932 in the training and validation sets. The AUC of the radiomics model was 0.937 (95% CI: 0.885-0.989) in the training cohort and 0.932 (95% CI: 0.860-1.000) in the external validation cohort, respectively. The levels of B3GALT4 mRNA were significantly different between the ALNM and non-ALNM groups (P<0.001). The clinical model achieved a higher AUC (training group, 0.904; validation group, 0.887). The nomogram performed well in both the training set (AUC = 0.991) and the validation set (AUC = 0.975). The nomogram had satisfactory clinical utility.

**Conclusion:**

The nomogram constructed by ultrasound features and B3GALT4 of the primary tumor can be used as an effective tool for individualized prediction of ALNM in breast cancer.

## Introduction

Breast cancer is the most prevalent malignancy among women globally and has increased significantly in recent years (1). The axillary lymph nodes (ALN) are the most prominent site for breast cancer metastasis, with multiple studies indicating that patients with positive axillary lymph node involvement exhibit a 5-year disease-free survival rate that is 20% lower than that of patients with negative involvement (2). In addition, ALN status is a key prognostic factor in the treatment strategy for breast cancer because it influences the scope of surgical intervention and evaluates the need for chemotherapy or radiation. Consequently, the precise assessment of the ALN condition is essential.

In present clinical practice, axillary lymph node dissection (ALND) and sentinel lymph node biopsy (SLNB) are often used to assess ALN status. Despite being the most common axillary staging technique, SLNB has drawbacks, such as lymphoedema or arm numbness (3). Even if the false-negative rate is acceptable, axillary lymph node metastasis (ALNM) may have gone undetected in certain instances (7.8–27.3%) (4). ALND can accurately determine ALN status and remove metastatic lymph nodes. However, ALND may result in serious side effects that might impair quality of life, including arm lymphoedema and shoulder dyskinesia (5, 6). Hence, precise preoperative evaluation of ALN metastases becomes especially important for preventing needless surgeries and creating individualized treatment strategies.

Preoperative imaging, such as computed tomography, magnetic resonance imaging, positron emission tomography, ultrasound (US), and mammography, has become more important and widely used in assessing ALNM in patients with breast cancer (7–10). Ultrasound is more economical, harmless, and repeatable than other imaging modalities. Radiomics has made substantial improvements in the investigation of ALNM in breast cancer. Previous investigations have demonstrated that multiple ultrasound characteristics of the primary tumor are associated with ALNM, such as maximum diameter, lesion margin, and extended range of enhancement lesions (11–14). However, imaging alone is always unsatisfactory in terms of diagnostic performance, with low sensitivity or specificity.

A number of glycosyltransferases have been identified as important regulatory factors in a variety of malignancies, including breast cancer (15–17). The β-1,3-galactosyltransferase-4 (B3GALT4) gene, which belongs to the family of β-1,3-galactosyltransferase genes, is significantly overexpressed in a variety of malignant tumor tissues (18, 19). In breast cancer cells, inhibition of the Smad3/4 complex binding to the B3GALT4 promoter SBE can lead to the down-regulation of the B3GALT4 gene expression, which in turn hinders the epithelial-mesenchymal transition process in breast cancer cells (20). Additionally, our previous study has shown that B3GALT4 was markedly overexpressed in breast cancer tissues and had a strong correlation with certain characteristics of clinicopathological status and unfavorable prognosis (21). Therefore, B3GALT4 is strongly linked with the progression of breast cancer.

Although nomogram models that incorporate ultrasound features for predicting ALNM have been widely researched, there is a paucity of studies that consider the integration of gene expression and ultrasound characteristics of primary tumors. The current study aimed to incorporate the ultrasonic features of primary tumors and the expression of B3GALT4 in tumor tissues to develop a model to predict ALNM in patients with breast cancer. After collecting ultrasound features and B3GALT4 mRNA expression data of breast cancer tissues, we would construct a combined model using machine learning and radiomics approaches. We sought to assess our model’s accuracy and dependability by comparing and validating it against real ALN status. This will help surgeons make better, more evidence-based therapeutic decisions.

## Materials and methods

### Data acquisition

**Figure 1:**
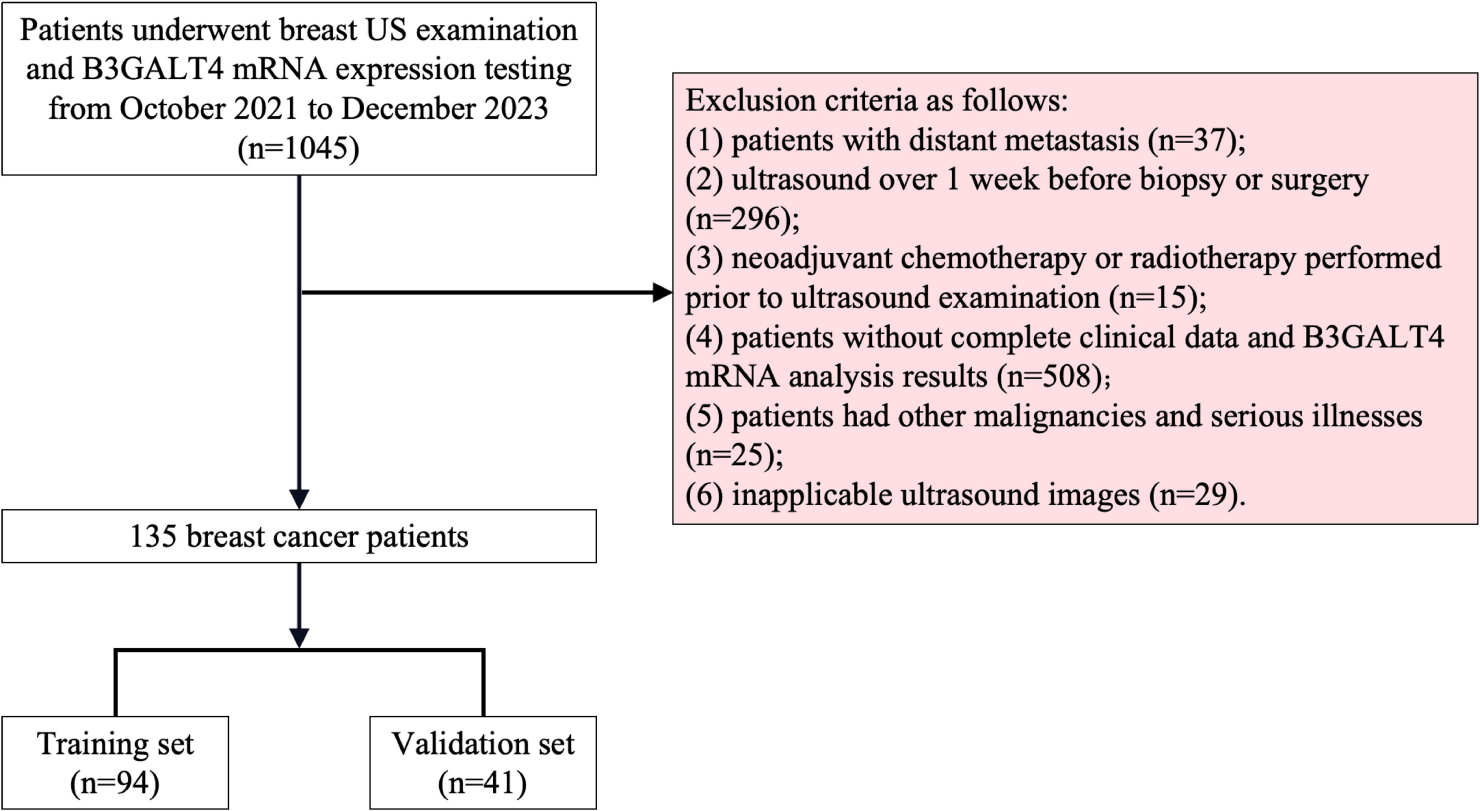
Recruitment scheme for patients in this study. 1045 breast cancer patients who received breast US examination and B3GALT4 mRNA expression testing were recruited. Based on the exclusion criteria, a total of 135 patients were included, and these patients were divided into training set (n=95) and validation set (n=40) in a 7:3 ratio. B3GALT4, Beta-1, 3-galactosyltransferase.

All the data were obtained according to the STROBE standards. The Ethics Committee of Xuzhou Central Hospital approved this research. Our study included 1045 breast cancer patients treated with ALND at Xuzhou Central Hospital from October 1, 2021 to December 31, 2023. The criteria for exclusion were listed below: (1) patients with distant metastasis; (2) ultrasound over 1 week before biopsy or surgery; (3) neoadjuvant chemotherapy or radiotherapy performed prior to ultrasound examination; (4) patients without complete clinical data and B3GALT4 mRNA analysis results; (5) patients had other malignancies and serious illnesses; (6) inapplicable ultrasound images. Finally, 135 breast cancer patients who met the criteria were included. **Figure 1** illustrates the patient recruitment procedure. The sample size estimation was performed based on results of previous related studies. In this study, a power value (probability of correctly rejecting a false null hypothesis) of 0.8 was chosen given a type I error rate of α = 0.05, and the effect size was set to 0.4. Based on the above sample size calculation formula and parameters, the estimated minimum sample size to obtain sufficient test power was 120. Yet, the sample size was increased to 135 to improve the power of the study.

### Ultrasound image acquisition

This research used ultrasound diagnostic devices like the PHILIPS EPIQ 5, GE LOGIQ E9, and SIEMENS ACUSON S2000. The probe models consisted of L12-3 (PHILIPS EPIQ 5), ML6-15-D (GE LOGIQ E9), and 14L5 (SIEMENS ACUSON S2000). For analysis, the patients were positioned supine with both arms elevated, thus completely exposing the breasts and axillary regions. Longitudinal, transverse, and radial scans focusing on the nipples were performed to assess both breasts. A scan of both axillary areas was conducted. The pictures were acquired in DICOM format. The US analysis were conducted by two professional sonologists who were not informed of the pathological information. The intra-class correlation coefficient (ICC) was conducted to assess the consistency between the two observers in analyzing radiomics features. Only the features with good consistency (ICC > 0.75) were selected for further analysis.

### Analysis of B3GALT4 mRNA level in breast cancer tissues

The researchers collected 135 patients’ fresh breast cancer samples. Quantitative real-time PCR (qRT-PCR) was used to analyze B3GALT4 mRNA expression in breast cancer tissues. First, we followed the manufacturer’s protocols to extract total RNA using TRIzol reagent (Invitrogen, USA). Then, we transcribed it to cDNA using a commercial reverse transcription supermix (Bimake, USA). Finally, we quantified it using SYBR The 2xSG Fast qPCR Master Mix (Sangon Biotech, China) on the Bio-Rad CFX96 machine. An internal reference known as GAPDH was used to normalize the mRNA levels. To calculate the relative expression, the 2^-ΔΔCt^ technique was used. This is a list of the B3GALT4 primer sequences: Forward: 5′-CTCCTGGCGGTCCTACTACT-3′, Reverse: 5′-CCACCACAGGCATGAGAGTT-3′, and the following were the GAPDH primer sequences: Forward: 5′-GGTATGACAACGAATTTGGC-3′, Reverse: 5′-GAGCACAGGGTACTTTATTG-3′.

### Image processing, segmentation, and feature extraction

We manually identified a rectangular region of interest (ROI) on the ultrasound image using the ITK-SNAP tool (21). The ROI involved the entire tumor area, including the complete hypoechoic tumor region, any echogenic halo, and other hypoechoic tumor regions. To ensure consistent voxel spacing, all pictures were adjusted to 1x1x1 mm. Ultimately, z-score standardization was used to normalize the data.

PyRadiomics is an open-access software platform designed for the extraction of features from medical pictures (22). The procedure included the manual importation of delimited ROI pictures into the PyRadiomics platform. The radiomic characteristics were categorized into three groups: geometry, intensity, and texture. Texture characteristics are retrieved through multiple techniques, including the gray-level co-occurrence matrix (GLCM), gray-level run length matrix (GLRLM), gray-level size zone matrix (GLSZM), and neighborhood gray-tone difference matrix (NGTDM). Z-score normalization was used to mitigate the problem of disparate scales in manual radiomic features.

### Feature selection and radiomics model construction

For every radiomic feature, we employed feature selection and the Mann-Whitney U-test. Radiomic features were only kept unless their corresponding P value was less than 0.05. Spearman correlation analysis was conducted on characteristics exhibiting high repeatability, and the correlation coefficients were then calculated. If the correlation coefficient between any two characteristics exceeded 0.9, only one feature was preserved. We employed the minimal redundancy maximum relevance (mRMR) technique to select the features that are most connected to ALNM. We further reduced the number of attributes needed to develop a signature by using the least absolute shrinkage and selection operator (LASSO) regression model. Using regulatory weight λ, LASSO minimizes regression coefficients to zero and properly adjusts many unnecessary attributes to zero. A 10-fold cross-validation with minimal criteria was used to determine the optimal λ value, resulting in the smallest cross-validation error. The chosen parameters with non-zero coefficients were merged into a radiomics signature.

We incorporate the final features from Lasso feature selection into various machine learning models such as Logistic Regression (LR), Naive Bayes, k-nearest neighbors (KNN), Decision Tree, Random Forest, Extra Trees, XGBoost, Support Vector Machine (SVM), and Multi-Layer Perception (MLP) to develop a model.

### Clinical model and radiomics-clinical nomogram model construction

Our previous research has shown a substantial correlation between the expression of the B3GALT4 gene in tumor tissues and axillary lymph node metastases in breast cancer patients (23). A clinical model was established based on the level of B3GALT4. A nomogram was constructed by combining B3GALT4 and the radiomics signature.

### Model evaluation

Every model received independent validation in both the testing and validation cohorts. Receiver operating characteristic (ROC) curves were constructed to visually assess each model’s diagnostic performance. The corresponding area under the curve (AUC), diagnostic accuracy, sensitivity, specificity, positive predictive value (PPV), and negative predictive value (NPV) were then analyzed to identify each model’s diagnostic efficacy.

**Figure 2:**
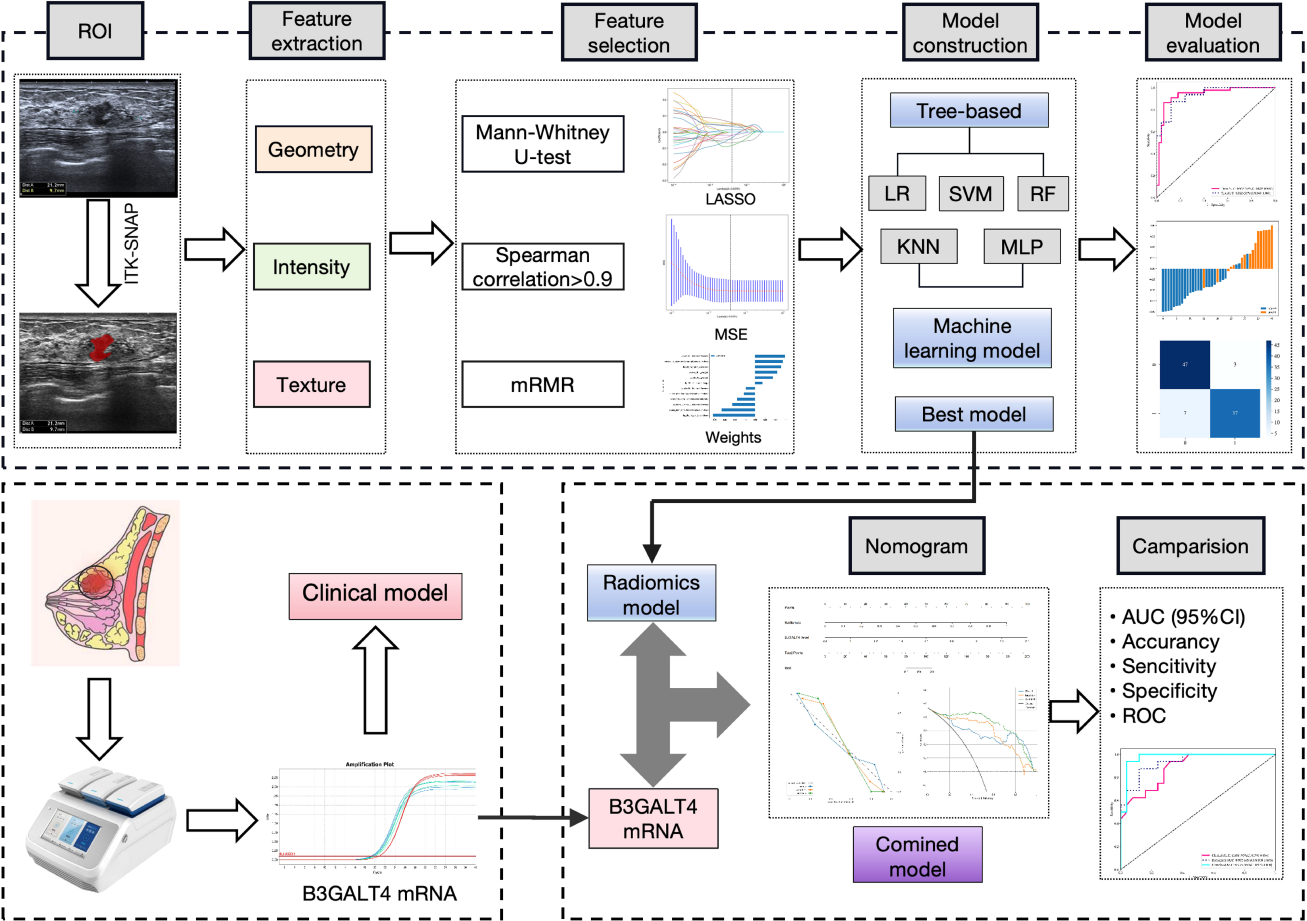
Workflow of this study. 1562 features were extracted from the ROI on the breast cancer ultrasound image of each patient. 12 features were refined using LASSO regression screening to construct a radiomics score. Consequently, linear-SVM was selected to develop a radiomics model. QRT-PCR was used to analyze B3GALT4 mRNA expression in breast cancer tissues. A clinical model was established based on the level of B3GALT4. Finally, a combined model was generated using the radiomics and clinical models, which was visualized using a nomogram. The model’s diagnostic performance was evaluated by the ROC curve. ROI, Rectangular region of interest; LASSO, Least absolute shrinkage and selection operator; qRT-PCR, Quantitative real-time polymerase chain reaction; ROC, Receiver operator characteristic.

The conformity between the estimated and true status of axillary lymph nodes was evaluated using calibration curves. The variation between the predicted and actual results was evaluated using the Hosmer-Lemeshow test. The nomogram’s practical value was evaluated using the decision curve analysis (DCA). The procedure of this study is shown in **Figure 2**.

### Statistical analysis

Statistical analysis was performed utilizing Python (version 3.70). Student’s t-test or Mann-Whitney U-test was used to evaluate continuous variables. The Chi-square test or Fisher’s exact test was used to evaluate categorical variables. A two-sided P-value of <0.05 was established to indicate statistical significance.

## Results

### Patient characteristics

The clinical characteristics of all included patients are shown in **Table 1**. All 135 patients were randomly divided into the training cohort (n=94) and the validation cohort (n=41) in a 7:3 ratio. There were no substantial differences in the US features, clinicopathological indicators, and B3GALT4 levels between the two groups.

**Table 1:** Patient characteristics across different cohorts.

Characteristic	Training Cohort (n=94)	Validation Cohort (n=41)	P
Clinicopathological characteristics			
Age	55.64 ± 11.21	55.79 ± 10.18	0.942
BMI(kg/m^2^)	24.20 ± 3.70	24.60 ± 3.40	0.555
SLNM			0.645
Positive	43(45.7)	17(41.5)	
Negative	51(54.3)	24(58.5)	
Pathology			0.981
IDC	64(68.1)	28(68.3)	
Others	30(31.9)	13(31.7)	
ER			0.663
Positive	61(64.9)	25(61.0)	
Negative	33(35.1)	16(39.0)	
PR			0.976
Positive	53(56.4)	23(56.1)	
Negative	41(43.6)	18(43.9)	
Her-2			0.991
Positive	32(34.0)	14(34.1)	
Negative	62(66.0)	27(65.9)	
Ki-67			0.604
<14	27(28.7)	10(24.4)	
≥14	67(71.3)	31(75.6)	
Histological grade			0.449
I-II	57(60.6)	22(53.6)	
III	37(39.4)	19(46.3)	
Tumor size			0.352
T1	54(57.4)	20(48.8)	
T2	40(42.6)	21(51.2)	
Ultrasound features			
Quadrant			0.342
Inner	71(75.5)	34(82.9)	
Outer	23(24.5)	7(17.1)	
Shape			0.915
Regular	5(5.3)	2(4.9)	
Irregular	89(94.7)	39(95.1)	
Margin			0.813
Smooth	3(3.2)	1(2.4)	
Non-smooth	91(96.8)	40(97.6)	
Calcifcation			0.590
No	46(48.9)	18(43.9)	
Yes	48(51.1)	23(56.1)	
Echo			0.354
Mixed	12(12.8)	3(7.3)	
Low	82(87.2)	38(92.7)	
BI-RADS category			0.744
4a	11(11.7)	4(9.8)	
4b or 4c	65(69.1)	31(75.6)	
5	18(19.2)	6(14.6)	
Longitudinal to transverse ratio			0.813
<1	3(3.2)	1(2.4)	
≥1	91(96.8)	40(97.6)	
B3GALT4 mRNA level	1.67 ± 0.52	1.65 ± 0.46	0.798

### Construction of radiomics model

**Figure 3:**
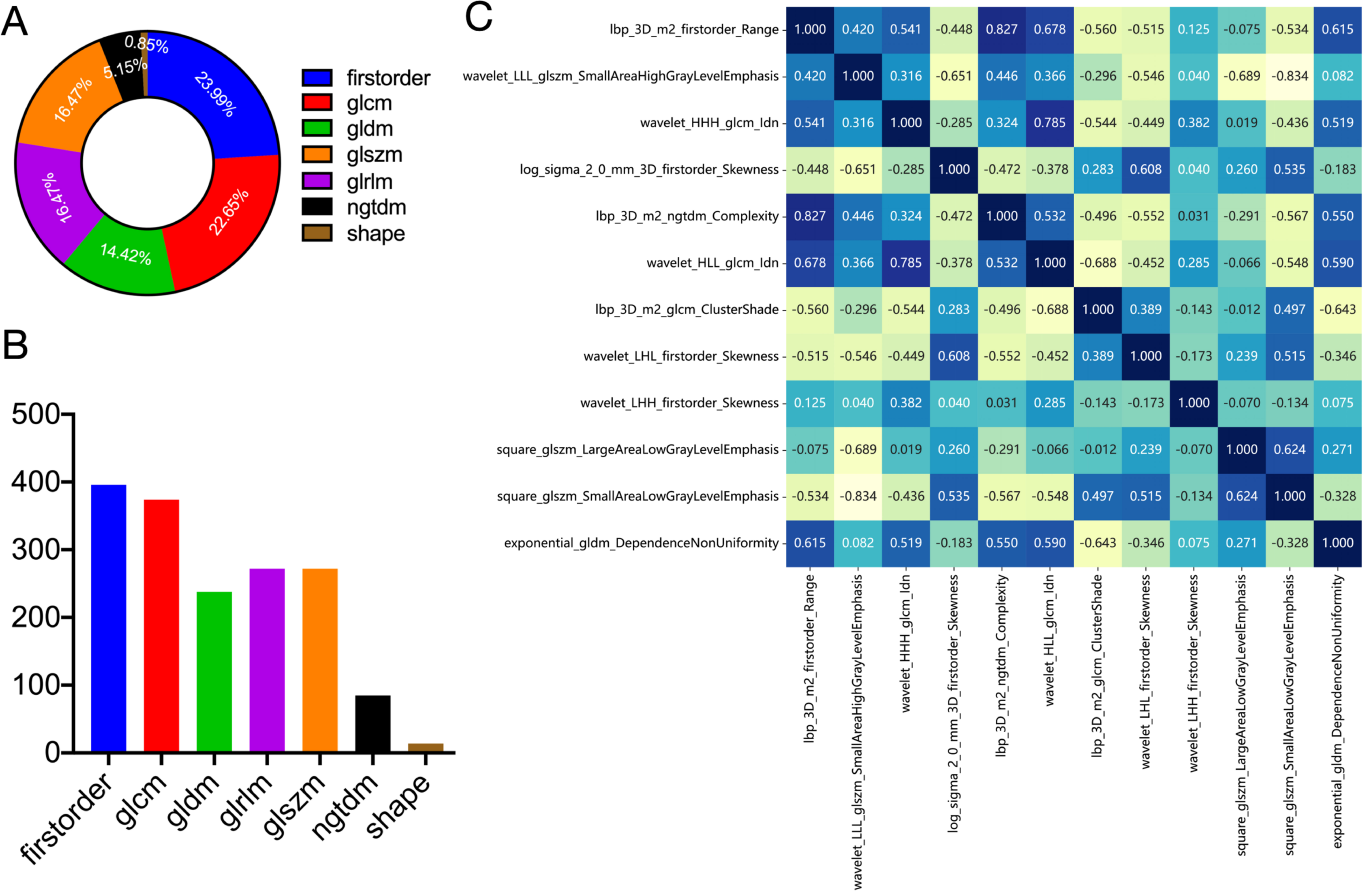
Definitions of radiomic features used in this study. Ratio **(A)** and number **(B)** of handcrafted features. **(C)** Spearman correlation coefficients between each feature. firstorder, first order features; glcm, gray level co-occurrence matrix features; gldm, gray level dependence matrix features; glszm, gray level size zone matrix features; glrlm, gray level run length matrix features; ngtdm, neighboring gray tone difference matrix features; shape, shape features.

**Figure 4:**
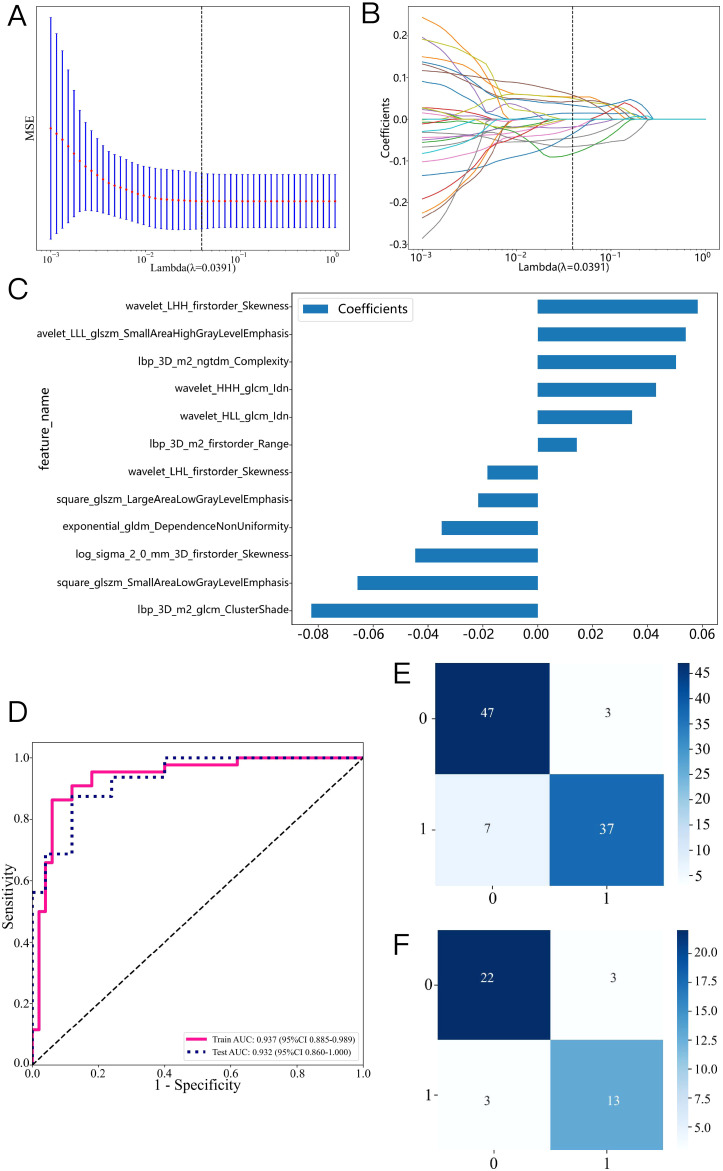
Radiomics feature selection based on the LASSO algorithm and construction of the radiomics model. **(A)**: the MSE of LASSO regression. **(B)**: the coefficients for cross-validation of LASSO regression. **(C)**: Selected features weight coefficients. **(D)**: ROC curve of the radiomics model in training and validation cohorts. Confusion matrix for the radiomics model in the training **(E)** and validation **(F)** cohorts.

1562 features were extracted from the ROI of each patient, including 306 first-order features, 14 shape features, and 1242 texture features. **Figures 3A, B** shows the amount and percentage of handcrafted characteristics. There was a total of 306 firstorder (23.99%), 374 glcm (22.65%), 238 gldm (14.42%), 272 glrlm (16.47%), 272 glszm (16.47%), 85 ngtdm (5.15%), and 14 shape (0.85%). A total of 1106 features exhibited significant differences between the ALNM and the non-ALNM groups. Afterwards, 216 features were retained for further investigation after a Spearman correlation analysis. Following validation using the mRMR method, only 30 features were preserved. 12 features with non-zero coefficients from the original set of 30 features were refined using LASSO regression screening to construct a radiomics score. Spearman correlation analysis showed that good agreement between each features (**Figure 3C**, coefficients -0.834 to -0.827). Subsequently, these features were evaluated using 10-fold cross-validation. Ultimately, LASSO regression model showed the best prediction performance when λ = 0.0391. **Figures 4A, B** represents the mean standard error (MSE) and the LASSO regression. The coefficient values of the non-zero characteristics were shown in **Figure 4C**. The calculation formula is as follows:

Label=0.4680851063829788 + 0.014251*lbp_3D_m2_firstorder_Range+0.053886*wavelet_LLL_glszm_SmallAreaHighGrayLevelEmphasis+0.043131*wavelet_HHH_glcm_Idn-0.044695*log_sigma_2_0_mm_3D_firstorder_Skewness+0.050389*lbp_3D_m2_ngtdm_Complexity+0.034417*wavelet_HLL_glcm_Idn-0.082545*lbp_3D_m2_glcm_ClusterShade-0.018325*wavelet_LHL_firstorder_Skewness+0.058408*wavelet_LHH_firstorder_Skewness-0.021728*square_glszm_LargeAreaLowGrayLevelEmphasis-0.065682*square_glszm_SmallAreaLowGrayLevelEmphasis-0.034928*exponential_gldm_DependenceNonUniformity.

A variety of machine learning models were developed and evaluated to identify the most effective model. **Supplementary Table S1** presents all models used in this study, revealing the linear-SVM model exhibiting superior performance relative to the other models. Linear-SVM had the highest AUC values in both the training (0.937, 95% CI: 0.885-0.989) and testing (0.932, 95% CI: 0.860-1.000) cohorts. Consequently, linear-SVM was selected as the basic algorithm to produce the radiomics scores. The optimal characteristics were integrated into the linear-SVM machine learning technique to develop a radiomics model via five-fold cross-validation. **Supplementary Figure S1** displays the sample prediction histogram of the SVM model. The blue section of the picture denotes individuals devoid of axillary lymph node metastasis, whereas the orange section denotes those with positive metastasis.

**Figure 4D** illustrates that the AUC of this model was 0.937 (95% CI: 0.885-0.989) in the training cohort and 0.932 (95% CI: 0.860-1.000) in the validation cohort. **Figures 4E, F** illustrate the confusion matrix of the radiomics model. The radiomics model had an accuracy of 0.894 (95% CI: 0.813-0.948), sensitivity of 0.841, specificity of 0.940, PPV of 0.925, and NPV of 0.870 in the training set. In the external validation cohort, the model attained an accuracy of 0.854 (95% CI: 0.708-0.944), sensitivity of 0.812, specificity of 0.880, PPV of 0.812, and NPV of 0.880.

### Construction of clinical model

**Figure 5:**
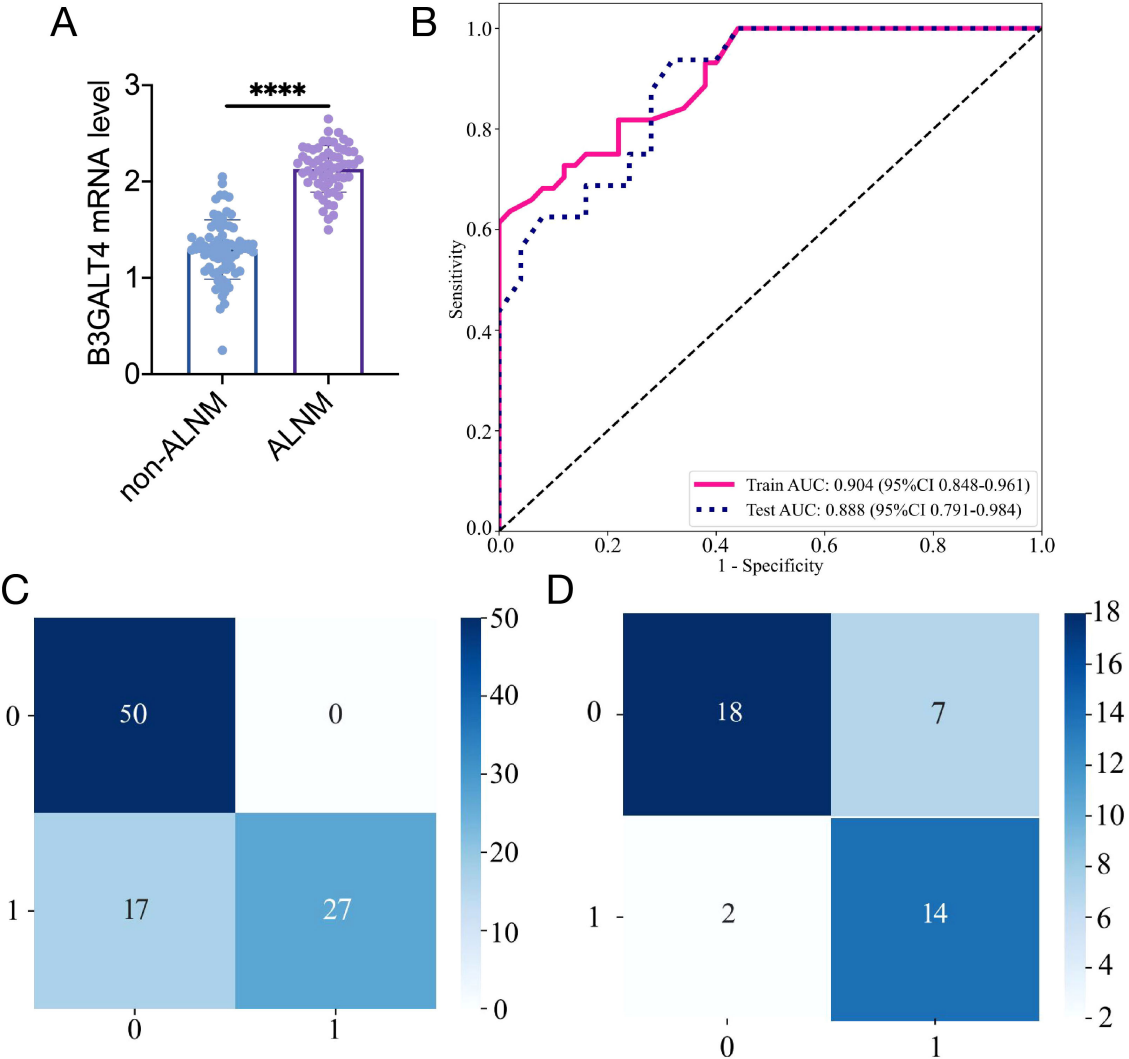
Construction of the clinical model based on B3GALT4 level. **(A)**: The mRNA level of B3GALT4 in the ALNM and non-ALNM groups. **(B)**: ROC curve of the clinical model in training and validation cohorts. Confusion matrix for the clinical model in the training **(C)** and validation **(D)** cohorts. ^****^ P<0.0001.

The levels of B3GALT4 mRNA were significantly lower in the non-ALNM group (1.30 ± 0.31) than the ALNM group (2.13 ± 0.24) (**Figure 5A**, P<0.001). Consequently, the SVM method was used to construct a clinical model using B3GALT4. The clinical model’s sample prediction histogram is represented in **Supplementary Figure S2**. The blue section of the picture denotes individuals devoid of axillary lymph node metastasis, whereas the orange section denotes those with positive metastasis.

The clinical model showed an AUC of 0.904 (95% CI 0.848-0.961), accuracy of 0.819 (95% CI: 0.726-0.891), sensitivity of 0.614, specificity of 1.000, PPV of 1.000, and NPV of 0.746, respectively, in the training cohort. In the test cohort, the model achieved an AUC of 0.887 (95% CI 0.791-0.984), an accuracy of 0.780 (95% CI: 0.624-0.894), sensitivity of 0.875, specificity of 0.720, PPV of 0.667, and NPV of 0.900 (**Figure 5B**). The clinical model’s confusion matrix is illustrated in **Figures 5C, D**.

### Construction of a combined nomogram model

**Figure 6:**
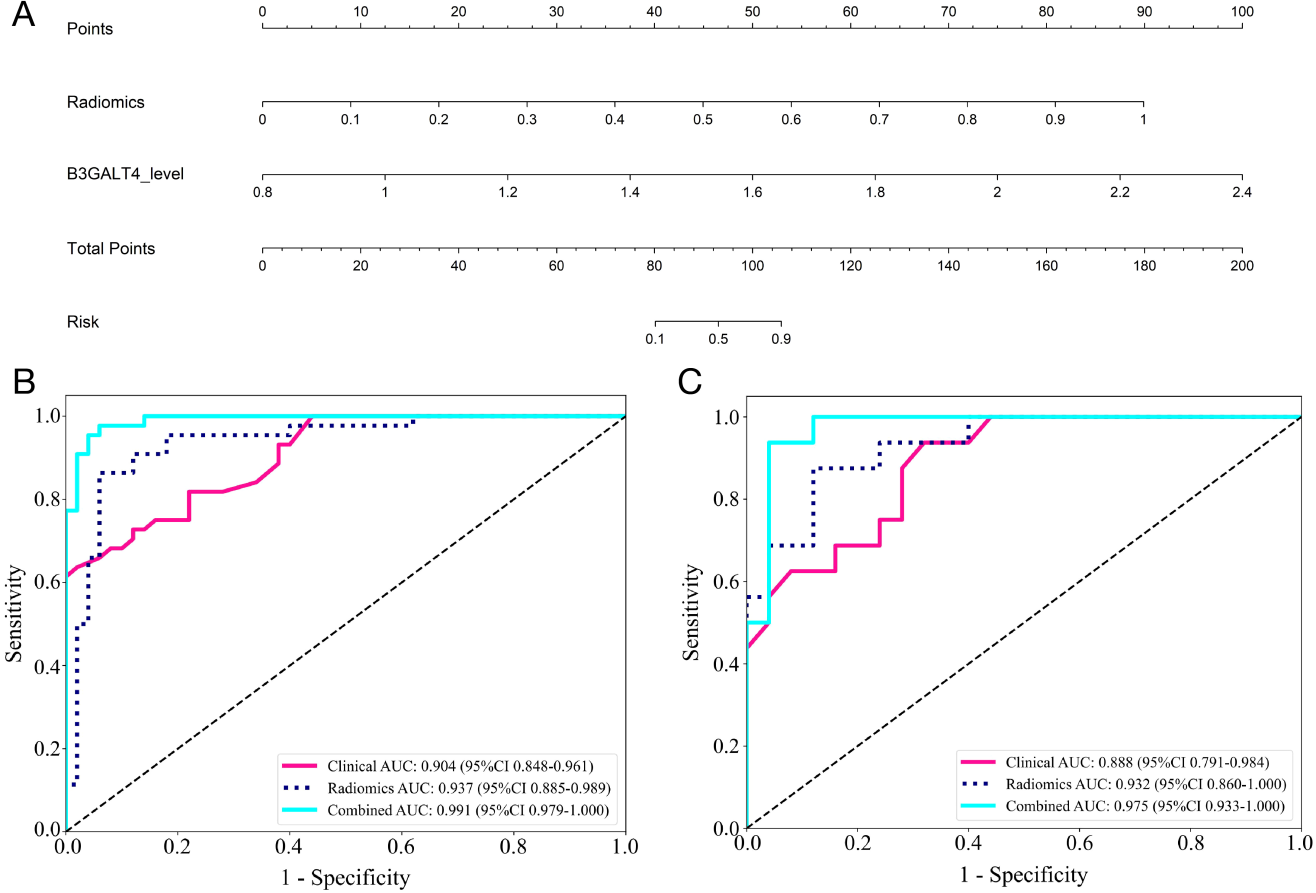
Comparison of the efficiency of the clinical, radiomic, and nomogram models. **(A)** The radiomics-clinical nomogram to predicting ALNM in breast cancer. The ROC curves and AUC of the clinical, radiomic, and nomogram models in the training **(B)** and validation **(C)** cohorts.

A combined model was generated using the radiomics and clinical models, which was visualized using a nomogram (**Figure 6A**). The diagnostic AUC, accuracy, sensitivity, specificity, PPV, and NPV of the combined model were 0.991 (95% CI: 0.979-1.000), 0.947 (95% CI: 0.880-0.983), 0.955, 0.94, 0.933, and 0.959, respectively, in the training cohort (**Figure 6B**). In the test cohort, the model achieved an AUC of 0.975 (95% CI 0.933-1.000), an accuracy of 0.927 (95% CI: 0.801-0.985), sensitivity of 0.875, specificity of 0.960, PPV of 0.933, and NPV of 0.923 (**Figure 6C**).

**Figure 7:**
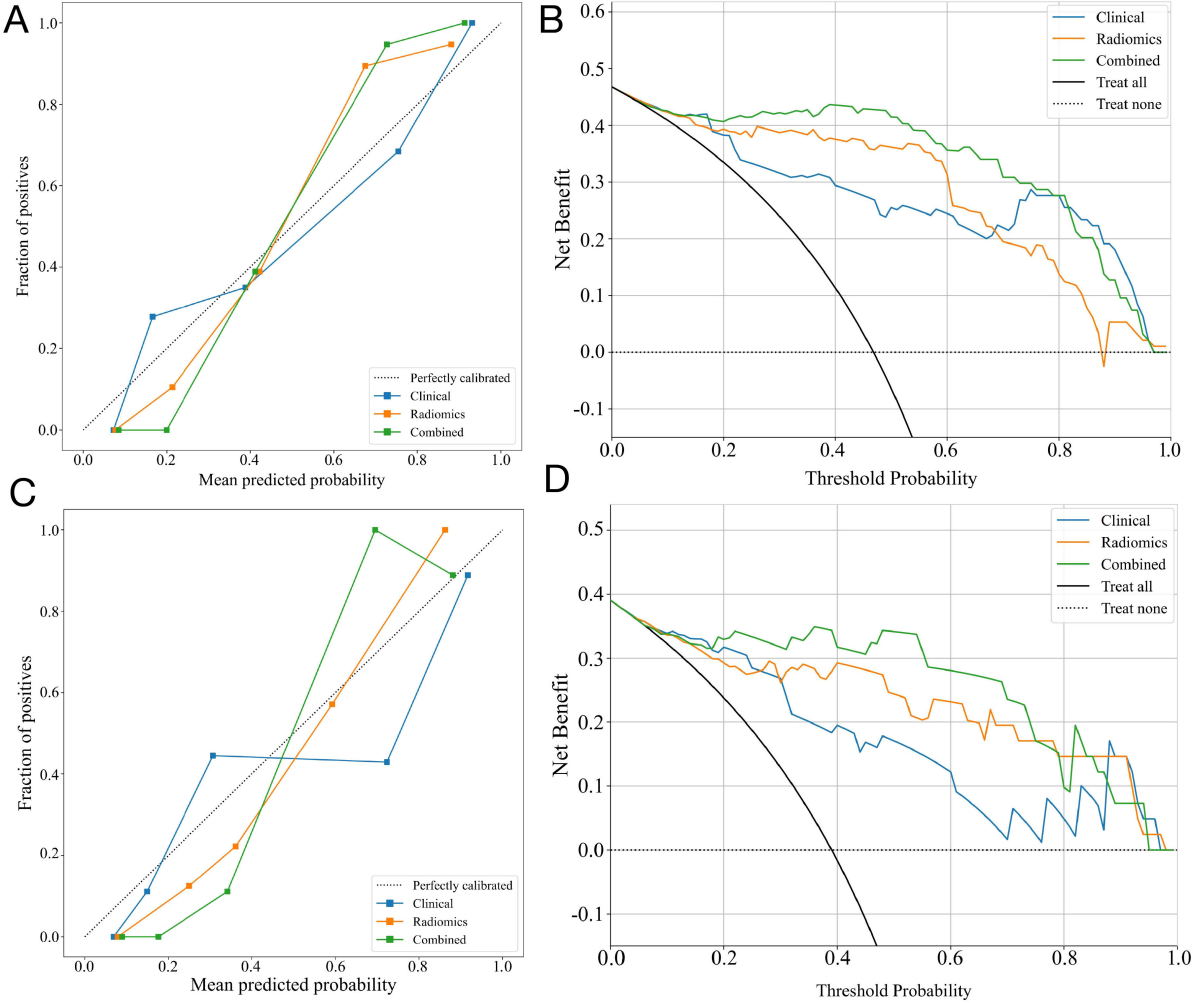
The performance of clinical, radiomic, and nomogram models in the training and validation cohorts. The calibration curves of three models in the training cohort **(A)** and the validation cohort **(C)**. The DCA curves for three models in the training cohort **(B)** and validation **(D)** cohorts show that the combined model has the greatest net benefit.

The calibration curves of the nomogram showed an excellent match of predicted ALNM with the true likelihood (**Figures 7A, C**). Moreover, the Hosmer-Lemeshow test revealed that the nomogram showed a strong fit (P = 0.173 in the training set; P = 0.082 in the validation set). DCA indicated that the combined model provided more net benefits than the radiomics and clinical models in predicting ALNM (**Figures 7B, D**).

## Discussion

Axillary lymph nodes are the predominant metastatic location for breast cancer. The condition of ALN is pivotal in determining the prognosis and therapeutic approach for breast cancer patients. Consequently, the precise prediction of ALNM and the identification of individuals who have an elevated axillary lymph node burden are both critical and challenging assignments. Although traditional US examination may detect markedly enlarged axillary lymph nodes and assess the likelihood for cancer metastasis based on morphology, margins, structure, and vascularity, it is not entirely reliable in predicting high-burden lymph nodes (24, 25). Herein, a prediction algorithm was developed by merging conventional ultrasound imaging with B3GALT4 analysis. Our findings indicated that this integrated model exhibits higher predictive efficacy in comparison with the single model, suggesting that it is helpful in assisting physicians in the selection of the most appropriate treatment. This study presents a trusted and feasible strategy for predicting the ALN status in breast cancer patients.

In recent years, with the advancement of radiomics and machine learning methods, a growing number of researchers have utilized these two methodologies in clinical imaging research (26, 27). For example, in experiments based on breast cancer ultrasound pictures, machine learning analysis enabled the development of a high-accuracy model for the identification of triple-negative breast cancer (AUC 0.88) (28). In predicting ALN metastasis in breast cancer, radiomics and machine learning methods have also shown excellent accuracy (29, 30). Qian et al. applied deep learning and ultrasound images of primary breast cancer to create a nomogram for assessing ALNM risk in breast cancer patients aged 75 years or older, with an excellent predictive accuracy (AUC 0.937) (31). Wu et al. exploited ultrasound-based radiomics and a deep-learning algorithm to identify ALN tumor burden in patients with node-positive breast cancer. The findings demonstrated that the machine learning model could identify the status of ALN tumor burden with higher accuracy and specificity than radiologists (32).

This study’s novelty is in the integration of radiomics and machine learning techniques to create an innovative model. The radiomics models proved to be effective in accurately distinguishing the status of ALNs. In the training cohort and test cohort, SVM obtained the highest AUC values of 0.937 and 0.932, respectively. In comparison to conventional image evaluation methods, the integrated model reveals exceptional accuracy and superiority by considerably increasing the AUC from 0.744 (previously reported) to 0.991 in predicting ALNM. The heterogeneity within tumors can be non-invasively captured using imaging omics technology. It utilizes sophisticated feature analysis algorithms to extract high-dimensional information from medical images. Conventional radiomics analysis mainly utilizes a single feature selection technique. In order to avoid overfitting, our work applies several machine learning techniques for feature selection first, followed by the application of LASSO for feature selection subsequently. The model not only gets higher predictive capacity but also offers interpretable results, which can assist clinicians in comprehending and using it.

To further improve the predictability, we combined B3GALT4 levels in tumor tissues with ultrasound imaging characteristics to create a comprehensive model. The results indicated that the integrated model had superior accuracy compared to the single-factor model. B3GALT4 is significantly overexpressed in many tumor tissues. B3GALT4 could inhibit the epithelial-mesenchymal transition in breast cancer cells (20). Moreover, our prior research demonstrated that B3GALT4 displayed elevated expression in breast cancer and was associated with tumor development. The negative predictive value achieved 0.959 in the training cohort and 0.923 in the test cohort, demonstrating a high degree of confidence in the model’s accuracy for predicting a negative ALN. This implies that the integrated model’s prediction of no axillary lymph node metastasis has excellent accuracy. The integrated model’s predictions of negative ALNs may provide doctors with a helpful reference, allowing them to avoid unnecessary ALN procedures and reduce the surgical risks. Alternatively, they may choose a fairly conservative non-surgical therapy strategy.

Our present study has several limitations. This is a retrospective, single-center research with a limited sample size; hence, a larger prospective investigation is required to further confirm its diagnostic efficiency. Secondly, despite the interpretation of ultrasound pictures by professional radiologists, a degree of subjectivity exists in evaluating ultrasound parameters, and the imaging characteristics analyzed in our research are limited. Consequently, a quantitative and effective approach to analyze conventional ultrasound images is very significant, such as integrating radiomics and deep learning to extract more informative ultrasound characteristics, which is the purpose of our further study. Moreover, future investigations should aim to clarify the mechanisms connecting B3GALT4 with ALNM, along with investigating novel treatment strategies.

## Conclusion

In summary, the nomogram combined ultrasound features with B3GALT4 of the primary tumor shows excellent accuracy and reliability in predicting the ALN status in breast cancer. This method may act as a significant reference for doctors to improve the effectiveness of personalized therapeutic strategies, assisting patients in avoiding unnecessary axillary lymph node surgery, thus minimizing surgical risks.

## Data Availability

The original contributions presented in the study are included in the article/**Supplementary Material**. Further inquiries can be directed to the corresponding author.
