# Supplementary material for: Ultrasound-based radiomics combined with B3GALT4 level to predict sentinel lymph node metastasis in primary breast cancer
Source: Front Oncol. 2025 Jul 11;15:1570493. doi: 10.3389/fonc.2025.1570493 (PMC12289485; doi:10.3389/fonc.2025.1570493)
Supplement: Supplementary file 1 [file DataSheet1.docx]

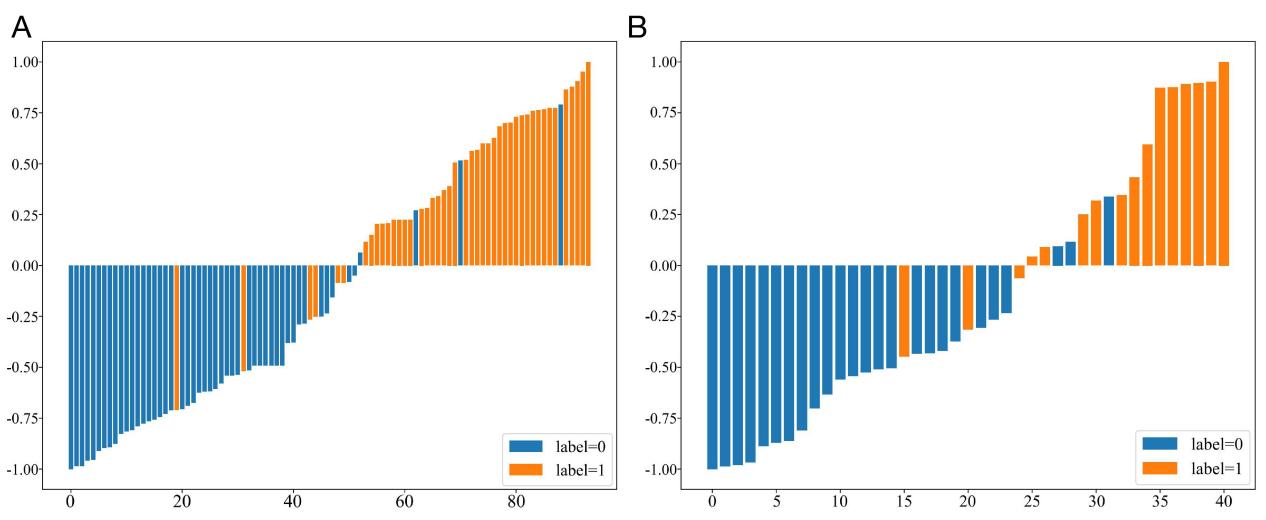


**Figure S1** Sample prediction histograms of the radiomics model for predicting ALN status in the training (A) and validation (B) cohorts. Label 1: ALNM; label 0: non-ALNM.


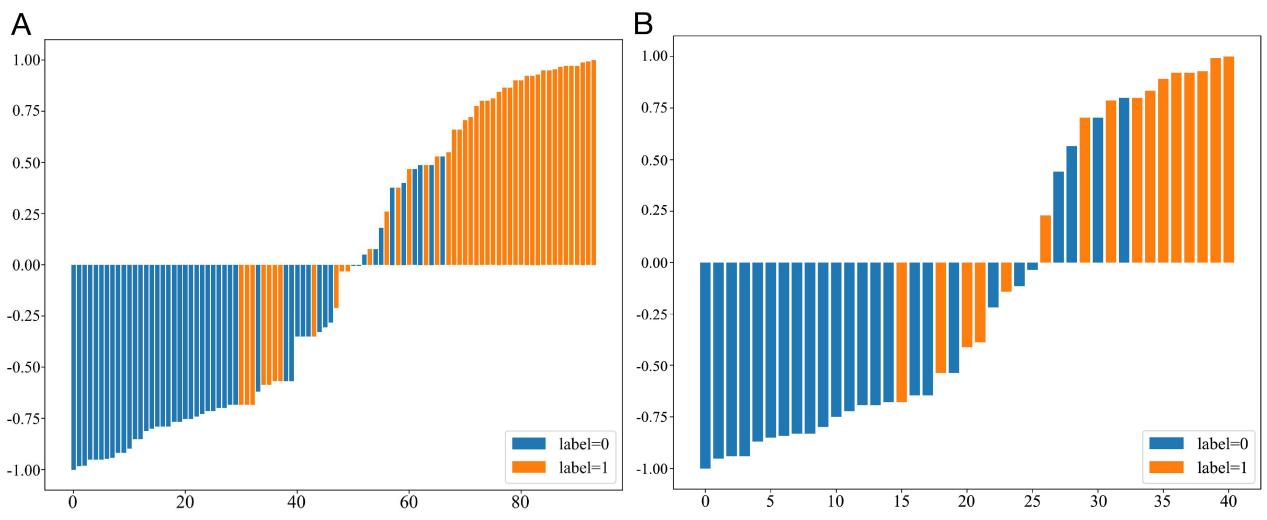


**Figure S2** Sample prediction histograms of the clinical model for predicting ALN status in the training (A) and validation (B) cohorts. Label 1: ALNM; label 0: non-ALNM.
