# Supplementary material for: Ultrasound-based radiomics combined with B3GALT4 level to predict sentinel lymph node metastasis in primary breast cancer
Source: Front Oncol. 2025 Jul 11;15:1570493. doi: 10.3389/fonc.2025.1570493 (PMC12289485; doi:10.3389/fonc.2025.1570493)
Supplement: Supplementary file 2 [file Table1.docx]

**Table S1** Diagnostic performance of different models.

| Cohort | Model | AUC | AUC 95%CI | Accuracy  (%) | Sensitivity  (%) | Specificity  (%) | PPV(%) | NPV(%) | Precision  (%) |
| --- | --- | --- | --- | --- | --- | --- | --- | --- | --- |
| Train | LR | 0.945 | 0.9021 - 0.9888 | 0.872 | 0.932 | 0.82 | 0.820 | 0.932 | 0.820 |
| Test | LR | 0.948 | 0.8870 - 1.0000 | 0.878 | 0.875 | 0.88 | 0.824 | 0.917 | 0.824 |
| Train | NaiveBayes | 0.931 | 0.8810 - 0.9808 | 0.851 | 0.909 | 0.8 | 0.800 | 0.909 | 0.800 |
| Test | NaiveBayes | 0.843 | 0.6971 - 0.9879 | 0.829 | 0.687 | 0.92 | 0.846 | 0.821 | 0.846 |
| Train | KNN | 0.936 | 0.8942 - 0.9785 | 0.766 | 0.5 | 1 | 1 | 0.694 | 1 |
| Test | KNN | 0.846 | 0.7304 - 0.9621 | 0.756 | 0.687 | 0.8 | 0.687 | 0.800 | 0.687 |
| Train | DecisionTree | 0.940 | 0.9044 - 0.9756 | 0.532 | 0 | 1 | 0 | 0.532 | 0 |
| Test | DecisionTree | 0.470 | 0.2881 - 0.6519 | 0.610 | 0 | 1 | 0 | 0.610 | 0 |
| Train | RandomForest | 0.982 | 0.9599 - 1.0000 | 0.936 | 0.977 | 0.90 | 0.896 | 0.978 | 0.896 |
| Test | RandomForest | 0.857 | 0.7436 - 0.9714 | 0.732 | 0.875 | 0.64 | 0.609 | 0.889 | 0.609 |
| Train | ExtraTrees | 0.936 | 0.8899 - 0.9828 | 0.872 | 0.864 | 0.88 | 0.864 | 0.880 | 0.864 |
| Test | ExtraTrees | 0.867 | 0.7585 - 0.9765 | 0.780 | 0.937 | 0.68 | 0.652 | 0.944 | 0.652 |
| Train | XGBoost | 0.994 | 0.9827 - 1.0000 | 0.968 | 0.977 | 0.96 | 0.956 | 0.980 | 0.956 |
| Test | XGBoost | 0.915 | 0.8327 - 0.9973 | 0.829 | 0.875 | 0.80 | 0.737 | 0.909 | 0.737 |
| Train | LightGBM | 0.959 | 0.9193 - 0.9993 | 0.915 | 0.864 | 0.96 | 0.950 | 0.889 | 0.950 |
| Test | LightGBM | 0.875 | 0.7714 - 0.9786 | 0.780 | 0.812 | 0.76 | 0.684 | 0.864 | 0.684 |
| Train | GradientBoosting | 0.998 | 0.9942 - 1.0000 | 0.979 | 0.955 | 1 | 1 | 0.962 | 1 |
| Test | GradientBoosting | 0.807 | 0.6696 - 0.9454 | 0.780 | 0.812 | 0.76 | 0.684 | 0.864 | 0.684 |
| Train | AdaBoost | 0.986 | 0.9667 - 1.0000 | 0.947 | 0.932 | 0.96 | 0.953 | 0.941 | 0.953 |
| Test | AdaBoost | 0.921 | 0.8438 - 0.9987 | 0.805 | 0.937 | 0.72 | 0.682 | 0.947 | 0.682 |
| Train | MLP | 0.943 | 0.8966 - 0.9898 | 0.904 | 0.864 | 0.94 | 0.927 | 0.887 | 0.927 |
| Test | MLP | 0.960 | 0.9092 - 1.0000 | 0.878 | 0.75 | 0.96 | 0.923 | 0.857 | 0.923 |
| Train | rbf_SVM | 0.943 | 0.8918 - 0.9946 | 0.904 | 0.909 | 0.90 | 0.889 | 0.918 | 0.889 |
| Test | rbf_SVM | 0.905 | 0.8168 - 0.9932 | 0.805 | 0.875 | 0.76 | 0.700 | 0.905 | 0.700 |
| Train | linear_SVM | 0.937 | 0.8848 - 0.9888 | 0.894 | 0.841 | 0.94 | 0.925 | 0.870 | 0.925 |
| Test | linear_SVM | 0.932 | 0.8603 - 1.0000 | 0.854 | 0.812 | 0.88 | 0.812 | 0.880 | 0.812 |
| Train | sigmoid_SVM | 0.924 | 0.8694 - 0.9779 | 0.862 | 0.841 | 0.88 | 0.860 | 0.863 | 0.860 |
| Test | sigmoid_SVM | 0.982 | 0.9545 - 1.0000 | 0.902 | 0.937 | 0.88 | 0.833 | 0.957 | 0.833 |
| Train | poly_SVM | 0.959 | 0.9229 - 0.9953 | 0.904 | 0.909 | 0.90 | 0.889 | 0.918 | 0.889 |
| Test | poly_SVM | 0.915 | 0.8316 - 0.9984 | 0.829 | 0.875 | 0.80 | 0.737 | 0.909 | 0.737 |
| Train | LDA | 0.939 | 0.8933 - 0.9849 | 0.872 | 0.932 | 0.82 | 0.820 | 0.932 | 0.820 |
| Test | LDA | 0.958 | 0.9052 - 1.0000 | 0.878 | 0.875 | 0.88 | 0.824 | 0.917 | 0.824 |
| Train | SGD | 0.933 | 0.8842 - 0.9813 | 0.872 | 0.886 | 0.86 | 0.848 | 0.896 | 0.848 |
| Test | SGD | 0.922 | 0.8424 - 1.0000 | 0.854 | 0.812 | 0.88 | 0.812 | 0.880 | 0.812 |
| Train | CatBoost | 0.989 | 0.9686 - 1.0000 | 0.968 | 0.955 | 0.98 | 0.977 | 0.961 | 0.977 |
| Test | CatBoost | 0.882 | 0.7819 - 0.9831 | 0.756 | 0.937 | 0.64 | 0.625 | 0.941 | 0.625 |
